# Not Whether, but in Whom: Reframing Antibody–Drug Conjugate Sequencing in Metastatic Breast Cancer

**DOI:** 10.1002/cai2.70085

**Published:** 2026-10-10

**Authors:** Armando Orlandi

**Affiliations:** ^1^ Comprehensive Cancer Center, UOC Medical Oncology Fondazione Policlinico Universitario Agostino Gemelli IRCCS Rome Italy; ^2^ Department of Translational Medicine and Surgery Università Cattolica del Sacro Cuore Rome Italy

## Abstract

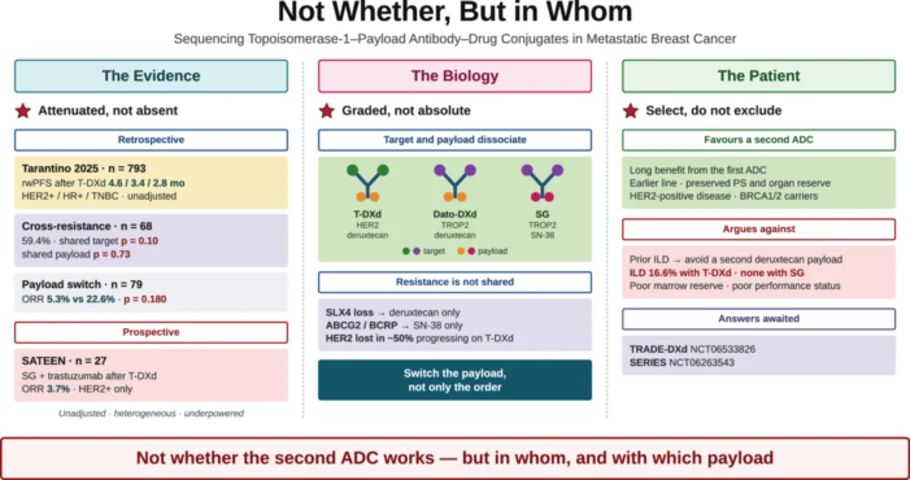

**Abbreviations**
ADC: antibody–drug conjugate
Dato‐DXd: datopotamab deruxtecan
ESMO: European Society for Medical Oncology
HER2: human epidermal growth factor receptor 2
HR: hazard ratio
ILD: interstitial lung disease
mBC: metastatic breast cancer
ORR: objective response rate
rwPFS2: real‐world progression‐free survival on the second
SG: sacituzumab govitecan
T‐DM1: trastuzumab emtansine
T‐DXd: trastuzumab deruxtecan
TOP1: topoisomerase I
TROP2: trophoblast cell‐surface antigen 2

1

Antibody–drug conjugates (ADCs) have redefined the treatment of metastatic breast cancer (mBC), delivering survival gains that surpass conventional chemotherapy across subtypes. Trastuzumab deruxtecan (T‐DXd) improved median overall survival by 6.4 months in human epidermal growth factor receptor 2 (HER2)–low disease, with an objective response rate of 52.6% in DESTINY‐Breast04, and sacituzumab govitecan (SG) extended survival in both triple‐negative and hormone receptor–positive disease [1, 2]. Success has created a new dilemma. Both agents deliver a topoisomerase I (TOP1)–inhibiting payload, and most patients are now candidates for both. Does the second ADC retain activity, or does shared‐payload cross‐resistance render direct sequencing futile? Two propositions can hold simultaneously: the activity of a second TOP1‐payload ADC is, on average, attenuated relative to its earlier use, and the evidence is nonetheless too weak to justify withholding it categorically. This commentary defends the second without disputing the first—a distinction that matters practically, because the answer is beginning to shape access and reimbursement decisions.

## 2 The Alert, and Its Fragility

The real‐world analysis by Tarantino et al. of 793 patients reported attenuated activity of the second ADC after T‐DXd, with SG ranking among the least active options [3]. The study is a substantial contribution, and its authors are appropriately measured; the restrictive reading it has attracted outruns what the data can bear. Comparisons are unadjusted, and the baseline imbalances are profound: patients receiving SG had the greatest prior‐treatment exposure—a median of four prior lines extending to twelve—with no reported data on performance status or disease burden, and no multivariable or propensity‐score adjustment. Channelling is the obvious alternative explanation: fitter patients receive chemotherapy, more heavily pre‐treated patients the second ADC. Most tellingly, within the same cohort SG was the most active regimen in triple‐negative disease, an internal contradiction that undercuts any blanket message of inefficacy.

## 3 A Heterogeneous, and Underpowered, Retrospective Landscape

Placed alongside its peers, the signal is harder still to read as a prohibition. Across retrospective series, the direction and magnitude of any sequence effect vary: some cohorts report no significant difference regardless of the order in which the two ADCs were given, while others find numerically better outcomes when SG precedes T‐DXd, without reaching significance [4]. In the largest dedicated cross‐resistance analysis, cross‐resistance to the second ADC occurred in 59.4% of evaluable cases and appeared graded according to whether target or payload was shared, yet neither comparison reached statistical significance (*p* = 0.10 and *p* = 0.73); the same held in the most cited payload‐switching series (*p* = 0.18) (Table 1) [4]. These are hypothesis‐generating observations from small, heterogeneous cohorts—a legitimate basis for caution and for trials, not for denying therapy.

**Table 1:** Key evidence on direct sequencing of topoisomerase‐1–payload ADCs in metastatic breast cancer, and its bearing on a restrictive rule.

Study (design, *n*)	Sequence	Key finding	Bearing on restriction
Tarantino 2025 (real‐world, 793)	After T‐DXd	rwPFS 4.6/3.4/2.8 months in *HER2*‐positive, HR‐positive, triple‐negative disease; SG among least active options; comparisons unadjusted	Hypothesis‐generating; channelling and baseline imbalance unaddressed
Mai 2025 (real‐world clinicogenomic, 85)	SG ↔ T‐DXd	rwPFS2 shorter in 75%, but 16% exceeded 6 months; PTEN loss predicted resistance to T‐DXd (HR 3.20) but not SG (HR 1.18)	Supports biomarker‐based selection, not exclusion
A3 cross‐resistance analysis (68)	Any two ADCs	Cross‐resistance 59.4%; 78.5% vs. 53.1% if target shared (*p* = 0.10); 60% vs. 52.4% if payload shared (*p* = 0.73)	Gradient in direction only; not statistically significant
Payload‐switch series (79)	T‐DM1, T‐DXd, RC48, SG	ORR on second ADC 5.3% with similar payload vs. 22.6% with different payload (*p* = 0.18)	Favours payload switching; underpowered
French multicentre series (179)	SG ↔ T‐DXd	Median PFS2 2.7 months; primary resistance to second ADC in 54.4%	~45% not primarily resistant
SATEEN (phase II, 27)	SG + trastuzumab after T‐DXd	Confirmed ORR 3.7% in extensively pre‐treated *HER2*‐positive disease	Attenuation confirmed in one narrow setting only

Abbreviations: ADC, antibody–drug conjugate; HR, hazard ratio; ORR, objective response rate; PFS2, progression‐free survival on the second ADC; rwPFS2, real‐world PFS on the second ADC; SG, sacituzumab govitecan; T‐DM1, trastuzumab emtansine; T‐DXd, trastuzumab deruxtecan.

## 4 Searching for the Subgroup, Not the Verdict

Against this background, the study by Mai et al. is the most pragmatically informative analysis available, because it asked a better question [5]. Rather than seeking a general verdict, it set out to identify the subgroups in which sequencing does and does not work. Among 85 heavily pre‐treated patients with *HER2*‐low mBC, real‐world progression‐free survival on the second ADC (rwPFS2) was shorter in 75%—yet 16% achieved an exceptional rwPFS2 exceeding 6 months, and the authors concluded that sequential ADC therapy with TOP1 payloads ‘is a viable treatment strategy in *HER2*‐low mBC’. Their clinicogenomic analysis generated the most provocative hypothesis: PTEN loss was associated with de novo resistance to T‐DXd (hazard ratio [HR], 3.20) but not to SG (HR, 1.18), while longer benefit from the first ADC and an earlier line of therapy predicted greater benefit from the second. These observations warrant caution: the cohort is small, retrospective and selected on having received both agents, and the authors themselves present the findings as hypothesis‐generating. They do not establish a biomarker. They do indicate, however, the direction in which the question should be asked—from categorical to predictive: not whether the second ADC works, but in whom and with which payload, it might still work.

## 5 Cross‐Resistance: Graded Rather Than Absolute

The premise underlying the alert is that a shared payload produces near‐complete cross‐resistance. Neither the clinical nor the pharmacological evidence supports so absolute a claim. Clinically, as above, the gradient is real in direction but statistically fragile. Pharmacologically, the case is more consistent: the two agents appear less interchangeable than their common TOP1 mechanism suggests [6]. Their resistance determinants are largely distinct—loss of SLX4 impairs sensitivity to deruxtecan while leaving camptothecin‐class agents such as SN‐38 unaffected—and each payload is handled by different efflux machinery, ABCG2/breast cancer resistance protein (BCRP) mediating SN‐38 resistance specifically. Resistance is also context‐dependent: *HER2* expression is lost in roughly half of tumours progressing on T‐DXd, favouring an antigen switch in *HER2*‐low disease, whereas payload‐level resistance and efflux dominate in *HER2*‐addicted disease [7]. Together these observations suggest a working principle rather than a settled rule: the two ADCs are pharmacologically non‐redundant, cross‐resistance is likely partial and payload‐dependent, and switching the payload mechanism may matter more than the fact of sequencing itself.

## 6 *HER2*‐Low and *HER2*‐Positive Disease Behave Differently

This model also accounts for a subtype‐specific disparity that deserves emphasis. In *HER2*‐low disease, the first ADC substantially outperforms the second: antigen down‐regulation is common at progression, so an agent directed at a different antigen but carrying the same payload class confronts both a depleted target and a shared resistance mechanism. In *HER2*‐positive disease, activity appears better preserved, particularly when T‐DXd follows T‐DM1—a switch of payload class on a retained, amplified target. DESTINY‐Breast03 established the superiority of T‐DXd over T‐DM1 in the second line (12‐month progression‐free survival 75.8% vs. 34.1%; HR 0.28; objective response 79.7% vs. 34.2%), while separate randomised evidence documents the efficacy of T‐DXd administered after prior T‐DM1 [4]. The contrast reinforces the central argument: what governs the activity of a second ADC is less the fact of sequencing than whether resistance at the level of both target and payload has been circumvented.

## 7 *BRCA1/2* Carriers: A Biologically Distinct Group

One group merits particular consideration before any restrictive rule is applied. TOP1 inhibition kills by generating DNA double‐strand breaks whose repair depends on homologous recombination; cells deficient in homologous recombination, as in *BRCA1/2*‐mutant disease, are correspondingly hypersensitive to SN‐38—the same synthetic‐lethal logic underpinning ongoing SG–poly(ADP‐ribose) polymerase (PARP) inhibitor combination trials, which exploit precisely this interaction and may yield the first direct clinical evidence [4]. In *BRCA1/2* carriers, the biological expectation therefore favours retained rather than abolished sensitivity to a trophoblast cell‐surface antigen 2 (TROP2)–directed, SN‐38–bearing ADC. This hypothesis, while biologically compelling, awaits dedicated clinical confirmation and should not yet guide routine clinical decision‐making. It does, however, caution against assuming a priori that these patients cannot benefit from direct sequencing.

## 8 The Intercalation Question

A related practice deserves separate treatment: interposing chemotherapy between two ADCs. Two distinct propositions must be separated. The first is mandatory intercalation imposed on theoretical cross‐resistance grounds, for which supporting evidence is lacking—real‐world data show that intervening therapy did not improve rwPFS on the second ADC [4]—and which substitutes an agent that is, in randomised trials, less active than the ADC it displaces. The second is deliberate bridging for a defined clinical indication: rapidly progressive visceral disease requiring immediate control, a planned interval to allow recovery from toxicity or of organ function, or a delay imposed by drug access. In such circumstances, intervening therapy may be entirely appropriate and should not be conflated with routine intercalation. Current evidence, in short, does not support inserting chemotherapy by default; it does not argue against doing so when a specific clinical reason exists. Bridging as a resensitisation strategy remains a hypothesis worth testing prospectively.

## 9 Safety, Cumulative Toxicity and Who Should Not Be Sequenced

Efficacy is only half of the sequencing decision; the other half is whether a given patient can safely receive a second ADC. The agents carry largely non‐overlapping toxicities—interstitial lung disease (ILD) with T‐DXd, myelosuppression and diarrhoea with SG—and that asymmetry has direct consequences. In a multi‐institutional cohort treated with both in sequence, ILD occurred in 16.6% of those receiving T‐DXd and in none receiving SG, was fatal in 5% and arose at a median of 3.0 months; T‐DXd also required more dose reductions [4]. Toxicity therefore constrains particular sequences rather than sequencing as such. Prior T‐DXd–related ILD does not argue against a subsequent switch to SG; if anything, it argues for it, since SG carries no comparable pulmonary signal. The constraint applies instead to sequences pairing two agents that carry the deruxtecan payload: datopotamab deruxtecan (Dato‐DXd), which shares the payload of T‐DXd and a recognised, if less frequent, ILD risk, so a T‐DXd–to–Dato‐DXd sequence in either direction stacks two pulmonary hazards [8]. This is precisely the sequence TRADE‐DXd is testing prospectively, and no real‐world data yet describe Dato‐DXd given after another ADC. Cumulative marrow suppression and declining hepatic or renal reserve further narrow the therapeutic window, and performance‐status decline is itself a frequent reason a second ADC is never given. Sequential therapy therefore demands caution, or is inadvisable, in patients with inadequate marrow reserve, poor performance status or unresolved toxicity from the first agent—and, where a second deruxtecan‐payload agent is contemplated, in those with prior or active ILD or significant baseline pulmonary compromise (Table 2). In such patients the realistic choice is not which ADC to give but whether to give one at all, a different chemotherapy and best supportive care being the appropriate comparators. Conversely, the non‐overlapping profiles can be exploited deliberately: SG is often preferred as the first ADC in patients with pre‐existing lung disease, and T‐DM1 remains an option for those unable to tolerate T‐DXd [4].

**Table 2:** Safety, cumulative toxicity and factors identifying patients unsuitable for sequential antibody–drug conjugate therapy.

Consideration	Trastuzumab deruxtecan	Datopotamab deruxtecan	Sacituzumab govitecan	Implication for sequencing
Target antigen	HER2	TROP2	TROP2	Dato‐DXd and SG share the same target but differ in payload
Payload	Deruxtecan (TOP1i)	Deruxtecan (TOP1i)	SN‐38 (TOP1i)	T‐DXd and Dato‐DXd share the same payload but differ in target
Principal toxicity	Interstitial lung disease (ILD)/pneumonitis	ILD/pneumonitis, less frequent; stomatitis, ocular events	Neutropenia, diarrhoea	Pulmonary risk is shared by the two deruxtecan‐payload agents but not by SG
ILD in patients treated with T‐DXd and SG in sequence	16.6%; fatal in 5%; median onset 3.0 months	No sequencing data available after a prior ADC	Not observed	Prior T‐DXd–related ILD argues for, not against, a switch to SG
Cumulative organ effects	Pulmonary reserve	Pulmonary reserve	Marrow reserve; hepatic and renal function	A T‐DXd–to–Dato‐DXd sequence stacks two pulmonary hazards
Caution, or unsuitable for a second ADC	Prior or active ILD; significant baseline pulmonary compromise	Prior or active ILD; significant baseline pulmonary compromise	Inadequate marrow reserve; unresolved diarrhoea	Constraint is agent‐specific, not generic to sequencing
Patient‐level factors, independent of agent	Unresolved toxicity carried over from the previous ADC (pneumonitis, cytopenias, diarrhoea, stomatitis and neuropathy); performance status; organ function; disease burden and pace of progression; comorbidity and patient preference			These factors, rather than the ADC class itself, often decide the next step: A second ADC, a different chemotherapy or best supportive care

*Note:* Sequential‐cohort ILD data as reported in reference [4].

Abbreviations: ADC, antibody–drug conjugate; Dato‐DXd, datopotamab deruxtecan; ILD, interstitial lung disease; SG, sacituzumab govitecan; T‐DXd, trastuzumab deruxtecan; TOP1i, topoisomerase I inhibitor.

## 10 Why Premature Restriction Would Harm Patients

The first prospective signal points in the same direction without settling the matter: in the phase II SATEEN trial, SG plus trastuzumab after prior T‐DXd yielded a confirmed response rate of 3.7% among 27 extensively pre‐treated patients with *HER2*‐positive disease (NCT06100874) [9]. This is consistent with attenuation of a second TOP1 ADC in heavily pre‐treated, *HER2*‐addicted disease, but it does not address the far larger *HER2*‐low population, payload‐switching strategies or biomarker‐selected patients. Contemporary reviews converge: the evidence base is immature, a consistent minority of patients benefit from the second ADC, and sequencing should be individualised rather than restricted [4, 8]. The 2026 European Society for Medical Oncology (ESMO) Clinical Practice Guideline states that the optimal ADC sequence has not been established and should be discussed with the patient [10]. Meanwhile, progression in mBC is often rapid, and withholding a potentially active ADC in favour of a less effective intervening therapy risks attrition through performance‐status decline.

## 11 Implications, and a Look Ahead

The appropriate response is neither reflexive sequencing nor categorical restriction, but better patient selection. In practice, that selection rests today on variables already available at the bedside rather than on novel assays: the duration of benefit from the first ADC, among the strongest predictors in the only clinicogenomic series to examine it [5]; the line of therapy; residual toxicity and organ reserve; the pace and burden of disease; performance status and comorbidity; and, realistically, drug access and reimbursement [4]. These are the factors that should inform the decision now. Investigational tools may eventually refine it, though none is validated for this purpose: re‐biopsy at progression would still interrogate a single lesion at a single moment; antibody‐based positron emission tomography might add spatial breadth across all lesions, but no tracer has been tested as a sequencing tool; and serial circulating tumour DNA (ctDNA) could supply the temporal dimension both lack, having shown that acquired resistance to SG is polyclonal, arising at both the antigen (TACSTD2/TROP2) and payload (TOP1) level [8]. This is a research programme, not a clinical algorithm. Prospective trials are addressing the question directly. TRADE‐DXd (NCT06533826) evaluates T‐DXd and datopotamab deruxtecan in either order in *HER2*‐low and *HER2*−0 disease progressing on a prior ADC; because both agents carry the same deruxtecan payload, the design holds payload constant and isolates the antigen question—whether it is preferable to target *HER2* or TROP2 first—thereby testing directly whether an antigen switch alone is sufficient. SERIES (NCT06263543) examines SG after progression on T‐DXd in *HER2*‐low disease. Until they report, the most defensible position is a modest one: the retrospective signal is real, and the activity of a second ADC is probably attenuated, but neither observation is a sufficient basis for categorical exclusion. Sequential ADC therapy should remain available to appropriately selected patients—chosen on clinical grounds and on the balance of efficacy and toxicity—rather than withheld on the strength of population averages and non‐significant comparisons.

## Author Contributions

**Armando Orlandi:** conceptualization, data curation, investigation, methodology, resources, software, supervision, validation, visualization, writing– original draft, writing – review and editing.

## Funding

The author has nothing to report.

## Ethics Statement

The author has nothing to report.

## Consent

The author has nothing to report.

## Conflicts of Interest

The author declares advisory board participation, speaker's fees and consultant roles for AbbVie, Menarini, Novartis, Pfizer, Eli Lilly, Amgen, AstraZeneca, Daiichi Sankyo and Gilead, and travel support from Menarini, Gilead, Daiichi Sankyo, Novartis, Roche and Pfizer. The author maintained full independence in study design, data analysis and interpretation.

## Data Availability

Data sharing not applicable to this article as no datasets were generated or analysed during the current study.

## References

[1] S. Modi , W. Jacot , T. Yamashita , et al., “Trastuzumab Deruxtecan in Previously Treated HER2‐Low Advanced Breast Cancer,” New England Journal of Medicine 387, no. 1 (2022): 9–20, 10.1056/NEJMoa2202491. PMC10561652 35665782

[2] A. Bardia , S. A. Hurvitz , S. M. Tolaney , et al., “Sacituzumab Govitecan in Metastatic Triple‐Negative Breast Cancer,” New England Journal of Medicine 384, no. 16 (2021): 1529–1541, 10.1056/nejmoa2028485. 33882206

[3] P. Tarantino , D. Lee , J. Foldi , et al., “Outcomes of Subsequent Treatment Regimens After Trastuzumab Deruxtecan in Patients With Metastatic Breast Cancer,” JNCI: Journal of the National Cancer Institute 117, no. 11 (2025): 2327–2335, 10.1093/jnci/djaf220.40811144

[4] R. Pacholczak‐Madej , M. Meluch , and M. Püsküllüoğlu , “Sequencing of Antibody Drug Conjugates in Breast Cancer: Evidence Gap and Future Directions,” Biochimica et Biophysica Acta (BBA) ‐ Reviews on Cancer 1880, no. 4 (2025): 189369, 10.1016/j.bbcan.2025.189369. 40484047

[5] N. Mai , M. M. Klar Lieberman , E. Ferraro , et al., “Sequential Antibody–Drug Conjugate Therapy in Patients With Metastatic Breast Cancer Treated With Sacituzumab Govitecan and Trastuzumab Deruxtecan,” JCO Precision Oncology 9 (2025): e2400898, 10.1200/PO-24-00898. PMC13532763 40440568

[6] M. Del Re , E. Inglese , E. Bria , et al., “Sequential Use of Trastuzumab Deruxtecan and Sacituzumab Govitecan in Patients With Breast Cancer: A Pharmacological Approach to Support the Clinical Rationale,” Drugs 86, no. 6 (2026): 871–885, 10.1007/s40265-026-02289-y. PMC13179276 41931218

[7] W. Chen , A. Gupta , N. Mai , et al., “Trastuzumab Deruxtecan Resistance *via* Loss of HER2 Expression and Binding,” Cancer Discovery 16, no. 2 (2026): 235–249, 10.1158/2159-8290.CD-25-0647. PMC12631751 41212147

[8] R. Colombo , P. Tarantino , J. R. Rich , P. M. LoRusso , and E. G. E. de Vries , “The Journey of Antibody–Drug Conjugates: Lessons Learned From 40 Years of Development,” Cancer Discovery 14, no. 11 (2024): 2089–2108, 10.1158/2159-8290.cd-24-0708. 39439290

[9] A. G. Waks and S. T. Investigators , “SATEEN: Sacituzumab Govitecan Plus Trastuzumab After Prior Trastuzumab Deruxtecan in HER2‐Positive Metastatic Breast Cancer,” Proceedings of ESMO Breast Cancer 2026 (2026): Abstract LBA4, 10.1016/S2059-7029(26)01690-X.

[10] E. de Azambuja , C. H. Barrios , R. Bartsch , et al., “Metastatic Breast Cancer: ESMO Clinical Practice Guideline for Diagnosis, Treatment and Follow‐Up,” Annals of Oncology 37, no. 9 (2026): 1203–1219, 10.1016/j.annonc.2026.05.707. 42217581

